# Medication in nursing homes in Alsace: a preferential list of drugs obtained by consensus

**DOI:** 10.1186/2193-1801-3-413

**Published:** 2014-08-07

**Authors:** Sophia Hannou, Amélie Rousseau, Marie-Christine Rybarczyk-Vigouret, Bruno Michel

**Affiliations:** OMEDIT d’Alsace, Cité administrative Gaujot, 14, rue du Maréchal-Juin, F-67084 Strasbourg, France; Service de Pharmacie, Centre Hospitalier Universitaire Vaudois, 46, rue du Bugnon, CH-1011 Lausanne, Switzerland; Service de Pharmacie, Hôpitaux Universitaires de Strasbourg, Université de Strasbourg, Faculté de Pharmacie, Laboratoire HuManiS (EA 7308), 1, avenue Molière, BP 83 049, Strasbourg, Cedex F-67098 France

**Keywords:** Appropriate drugs, Elderly, Delphi method, Medication, Nursing home, Improving patient care, Optimizing prescription

## Abstract

**Abstract:**

In order to improve patient care, OMEDIT (Observatory of drugs, medical devices and therapeutic innovation) Alsace, conducted a study to develop a Preferential list of Drugs adapted to the Elderly (PDE list) in nursing homes. The study conducted from December 2011 to June 2012 was organized in 4 phases: 1) creation of a preliminary list of drugs from those currently used in nursing homes in Alsace, 2) application of a two-round Delphi process to evaluate the preliminary list involving mobilization of experts from different backgrounds (geriatricians, general practitioners, pharmacists …), 3) identification of molecules considered in literature as potentially inappropriate, 4) generation of a final PDE list, including information concerning proper use of drugs for prescription and administration. 53 experts participated in the study. In the first round, 338 drugs were on the preliminary list, 246 were considered as appropriate by experts and 28 as inappropriate. 64 drugs without consensus were submitted to a second round. 32 of them were considered as inappropriate and 32 others remained on the list with no consensus. These last 32 were evaluated by OMEDIT and 3 were considered as appropriate drugs for the elderly. Totally, 252 drugs constitute the final PDE list from our study. The PDE list constitutes a new guide for optimization of both prescription and administration of drugs in nursing homes and could help reduce misuses and poly-medication, which are constant preoccupations to avoid adverse drug reactions in elderly.

**Key points:**

● The study was carried out with the aim to create a Preferential list of Drugs adapted to the Elderly (PDE list) in nursing homes using a modified Delphi method.

● The PDE list constitutes a new guideline to harmonize practices in nursing homes and to help physicians and nurses to achieve best possible care management.

## 1. Introduction

The European population is living longer than before and the percentage of the elderly in society is a significant demographic change, and as a consequence, the part of this population going to nursing homes is growing. The health status of these frail and/or dependent patients with various pathologies often requires the use of several medications. In this regard, a number of studies have shown that poly-medication, defined as medication with at least 5 to 8 drugs, is common in the elderly with the highest number of drugs taken by those residing in nursing homes (Maher et al. 2014). With the use of multiple medications, an increased risk for negative health outcomes has been described (Maher et al. 2014).

Reducing overuse of drugs in nursing homes, especially to prevent adverse drug reactions (ADRs), is a major health concern and constitutes a challenge for every care giver, even for the most experienced clinicians (Petrovic et al. 2012). In the elderly, the risk of ADRs is positively correlated to the number of drugs used. As an illustration, elderly patients taking 2 drugs face a 13% risk of adverse drug-drug interactions, and this rises to 38% for 4 drugs and to 82% if 7 or more drugs are given simultaneously (Leendertse et al. 2008; Beijer & de Blaey 2002). The ADRs, in the elderly, often lead to hospitalization and increased expenditure for medical care (Chen et al. 2014). According to Beijer et al., the average rate of ADR-related hospital admissions is 16.6% in older patients, compared to 4.1% in younger patients (Beijer & de Blaey 2002).

Besides overuse, other considerations must be taken into account to render medications and health conditions secure in the elderly: misuse or under-use of drugs, respect of dosage, appropriate pharmaceutical form in case of deglutition problems. Other factors to be considered are impairment renal or hepatic function and the clinical characteristics of the patient such as alteration in cognitive function, urinary incontinence (Topinkova et al. 2012).

Physicians who are working in nursing homes are usually not geriatricians. Their workload mostly does not allow time for in-depth reviews of literature, which is currently starting to be rich in drug- safety specific reports and lists of potentially inappropriate medication (PIM) for the elderly. Considering the given situation, the idea of guiding the prescription to treat the main pathologies encountered towards drugs belonging to an arsenal validated by a panel of recognized experts was attractive.

This present study was carried out with the aim to create a preferential list of Drugs adapted to the Elderly (PDE list) in nursing homes using a modified Delphi method. This PDE list was based on personal experience/opinion of 53 experts and available evidence.

The final goal was to provide a support document to help health-care professionals (mainly general practitioners, but also nurses and pharmacists) in nursing homes to set their own guidelines for one main objective: simplifying prescription and administration in order to improve the management of drug therapy in the elderly.

## 2. Methods

This study was carried out from December 2011 to June 2012 and was conducted by OMEDIT (Observatory of drugs, medical devices and therapeutic innovation) Alsace in France. Alsace is a region in France with 1.8 million inhabitants.

The literature contains many different age thresholds for the definition of the elderly. The threshold of 65 years with poly-pathologies or 75 years or more were the cut-offs retained in this study [ANSM (ex Afssaps) 2005].

### Study design

**Figure 1:**
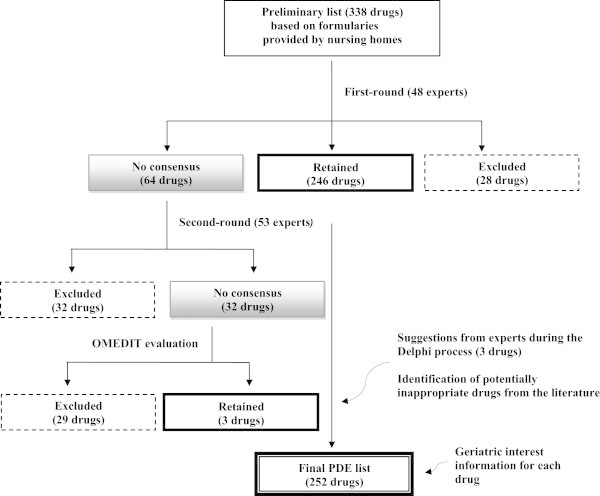
**Presentation of the Delphi process used to generate the preferential list of drugs adapted to the elderly (PDE list).**

The data was collected in 4 phases (Figure 1).

1. Creation of a preliminary list of drugs based on formularies used in daily practices in nursing homes in Alsace.
2. Application of a two-round Delphi process to evaluate the preliminary list in order to lead to build a PDE list. This step included:
  - recruitment of experts,
  - creation of a first-round of a survey and subsequently sending *e-*mails to the experts,
  - creation of the second-round of the survey based on results from the first round and receiving feedback from the panel of experts.
3. Identification of molecules considered in literature as potentially inappropriate in the preliminary list and in the PDE list obtained from Delphi process.
4. Generation of the final PDE list containing appropriate drugs for the elderly based on the experts’ opinion and combined also with practical information for the proper use of drugs, in terms of prescription and administration.

### 2.2. Creation of a preliminary list of drugs commonly used in nursing homes

An inventory of the available drug formularies used in nursing homes in Alsace was carried out in December 2011. Drugs present in more than 20% of the formularies analyzed were selected to be part of the preliminary list of the study. Specific treatments such as antineoplastic drugs were excluded from the study.

### 2.3. Application of the Delphi method

The Delphi method, developed by the Rand Corporation in the 1950s, is a research method allowing a consensus opinion to be reached among experts through an iterative and anonymous process known as rounds (Dalkey 1969). The method uses surveys in order to collect information. Two rounds were carried out in our study. The responses from the first round were collected and analyzed; a revised survey was then submitted to the experts to initiate the second round.

#### Experts: panel selection

After consulting several healthcare professionals and scientific societies specialized in the field, a panel of potential members with recognized expertise was identified and invited to participate in this study. Care was taken to select experts from Alsace (44 = experts) but also from other parts of France (n = 4 experts) and from neighboring countries (n = 5 experts). These experts represented 6 different specialties [geriatric medicine (n = 22), clinical pharmacology (n = 2), general practice in nursing homes (10), pharmacovigilance (n = 2) and pharmacy (n = 17 experts, 7 community pharmacists practicing in nursing homes and 10 hospital pharmacists].

#### Data collection and analysis

##### First-round

The preliminary list was used to formulate the first round of the survey which was then sent by e-mail to the experts. They rated each drug on a five-point Likert scale, which ranges from a score of 1 (drugs that can be considered as appropriate for the elderly in nursing homes) to 5 (drugs that can definitely be considered as inappropriate in nursing homes) (Matell & Jacoby 1971). An appropriate drug was defined as an indispensable drug with a clear-cut benefit in terms of efficacy/safety ratio or a drug with obvious efficacy.

A score of 3 was considered as neutral (undecided). After the first round, the mean Likert score and the corresponding 95% confidence interval (CI) were determined for each drug. Drugs, for which the upper bound of the 95% CI was less than 3.0, were classed as appropriate, while drugs, for which the lower bound of the 95% CI was greater than 3.0, were classed as inappropriate drugs for elderly persons. Only the drugs whose 95% CI was on both sides of 3.0, were evaluated further by the experts, in the second round of questioning. During the first-round, experts were also invited to add comments, to suggest safer or more appropriate alternative therapeutics.

##### Second-round

The second-round of the survey included drugs that didn’t reach consensus from round-one with the results of their scores and any statements added by the experts. The data were presented anonymously, enabling the participants to reconsider their previous responses. The answers provided by the experts in the second round were evaluated by the same procedure described above.

### 2.4. Potentially inappropriate medications in the elderly

Three lists of PIM were used to identify drugs categorized as inappropriate from the preliminary and the PDE lists (The American Geriatrics Society 2012 Beers Criteria Update Expert P 2012; Holt et al. 2010; Laroche et al. 2007). We had access to two European lists (one from Germany and one from France) and one list from the American geriatrics society. Although it was not specifically developed for European countries, the list from the United States of America (USA) has already been successfully used to detect PIM in European countries and it has been recently updated (Laroche et al. 2007).

### 2.5. Generation of the final PDE list

The final PDE list was established on the basis of two rounds of the Delphi process. Drugs considered as appropriate in nursing homes by experts but qualified as inappropriate by the literature have been nevertheless retained in the final PDE list. Information concerning the proper use of drugs was added for each drug in the list. Concerning this latter point, the data was taken from different sources: European Medicines Agency (EMA), French national agency of drug security (ANSM, Agence Nationale de Sécurité du Médicaments et des produits de santé), French database Thériaque (2014), OMEDIT Normandy (2014) and pharmaceutical companies.

### 2.6. Statistical analysis

Statistical calculations were performed with the SAS program, version 9.1 (SAS Institute Inc Cary, North Carolina, USA).

## 3. Results

Figure 1 summarizes the process used to generate the PDE list.

### 3.1. Creation of a preliminary list of drugs used in nursing homes

One hundred and five nursing homes of Alsace were asked whether they had a formulary of drugs in their facility. Only 23 nursing homes had formularies, of which 20 provided a copy of their document*.* After reviewing the 20 formularies, we found that they actually corresponded to 15 different lists of drugs. Indeed, in some cases, several nursing homes shared the same formulary. Of the 15 drug formularies, 11 were finally used for the design of the preliminary list. Three formularies were excluded because they were consumer reports without any indications to ensure the proper use of medications and one corresponded to a list of medications which had to be avoided in the elderly.

Drugs present in more than 20% of the 11 analyzed formularies were then chosen to be part of the preliminary list of our study. Three hundred and thirty eight drugs of the 591 on the lists were thus selected and the preliminary list of drugs was constituted. Table 1 shows the distribution of the 338 molecules of this list according to the ATC classification (Anatomic, Therapeutic and Chemical classification).

**Table 1:** Distribution of drugs in the preliminary list according to the ATC* classification

ATC classification system	Number of drugs
**A. Alimentary tract and metabolism**	54
**B. Blood and blood forming organs**	15
**C. Cardiovascular system**	59
**D. Dermatologicals**	26
**G. Genito urinary system and sex hormones**	12
**H. Systemic hormonal preparations**	9
**J. Anti-infective for systemic use**	29
**L. Antineoplastics and immunomodulating agents**	5
**M. Musculo-skeletal system**	20
**N. Nervous system**	74
**P. Antiparasitic products, insecticides and repellents**	0
**R. Respiratory system**	22
**S. Sensory organs**	13

*ATC: Anatomic, Therapeutic and Chemical classification.

### 3.2. Application of the Delphi method

Contacts were established with 50 experts, of whom 48 agreed to participate in the project. The 48 experts completed all rounds of the survey (from February to April 2012). Five additional experts, who were subsequently included by personal communication, then joined the study during the second-round. Finally, the panel of experts was composed of physicians, representing two-thirds of the panel and pharmacists the remaining one-third.

**Table 2:** **Drugs for which the experts did not reach a clear decision after the 2-round Delphi process**

ATC* classification system	Drug evaluation on the 5-point Likert scale mean, and 95% confidence interval
**A. Alimentary tract and metabolism**	
Glibenclamide	3,17 [2,88; 3,46]
Loperamide	3,12 [2,82; 3,41]
Mebeverine	3,15 [2,89; 3,41]
Metopimazine	3,10 [2,84; 3,36]
Miconazole	3,13 [2,85; 3,42]
Ornithine oxoglurate	3,31 [2,99; 3,64]
Porcine pancreatin	3,18 [2,97; 3,39]
Saccharomyces Boulardii	2,81 [2,50; 3,12]
Sitagliptin	3,17 [2,91; 3,44]
Trimebutine	2,96 [2,70; 3,23]
**C. Cardiovascular system**	
Bisoprolol + hydrochlorothiazide	3,12 [2,88; 3,35]
Spironolactone + furosemide	2,94 [2,67; 3,21]
**D. Dermatologicals**	
Dexpanthenol	3,08 [2,77; 3,39]
Retinol	3,02 [2,78; 3,26]
**J. Antiinfectives for systemic use**	
Fosfomycin	3,33 [2,98; 3,67]
Spiramycin + metronidazole	3,38 [2,52; 3,06]
**M. Musculo-skeletal system**	
Diclofenac	3,02 [2,70; 3,34]
Ibuprofen	3,38 [2,98; 3,60]
**N. Nervous system**	
Acetylleucine	3,02 [2,72; 3,32]
Betahistine	2,96 [2,66; 3,26]
Clonazepam	3,21 [2,91; 3,52]
Duloxetine	2,76 [2,51; 3,02]
Fluoxetine	3,18 [2,93; 3,43]
Haloperidol	2,88 [2,60; 3,16]
Hydroxyzine	3,08 [2,75; 3,40]
Lorazepam	3,13 [2,82; 3,44]
Lysine acetylsalicylate	3,24 [2,95; 3,53]
Milnacipran	2,96 [2,70; 3,21]
Tianeptine	3,24 [2,97; 3,50]
Tramadol	2,94 [2,67; 3,20]
Tramadol + acetaminophen	3,14 [2,86; 3,41]
**S. Sensory organs**	
Dexamethasone + neomycin + polymyxin B	2,90 [2,62; 3,18]

*ATC: Anatomic, Therapeutic and Chemical classification.

- Based on the results from the first round which evaluated 338 drugs: 246 molecules of the preliminary list were judged to be appropriate for the elderly, 28 drugs were excluded and a consensus could not be reached for 64 other drugs. Thus, a further evaluation was needed for these last 64 drugs.
- After the second round: 32 drugs were definitely considered as inappropriate for the elderly. For the last 32 drugs, no consensus was obtained (see Table 2). OMEDIT, identified as a group of experts, evaluated these latter 32 drugs.
- OMEDIT evaluation: 29 of the 32 drugs were excluded by OMEDIT Alsace, considering the existence of therapeutic alternatives validated by experts during the first round; the remaining 3 drugs [ibuprofen, diclofenac and tramadol] were on the other hand retained in the list.

During the Delphi process, the experts also suggested new drugs as possible appropriate medications. Among proposals made by the experts, 3 suggestions were retained by OMEDIT Alsace (an antiseptic mouthwash containing chlorhexidine and chlorobutanol, heparin calcium which is suitable for people with impaired renal function and a skin antiseptic containing chlorhexidine). Thus, the Delphi process finally identified 252 drugs. These constitute the proposed PDE list presented in the article.

### 3.3. Potentially inappropriate medications in the elderly

Of the 338 starting molecules (preliminary list of the study) and the 252 drugs of the PDE list, 68 (20%) and 30 (12%) were respectively considered as potentially inappropriate in the elderly according to the 3 PIM lists mentioned in the methods (The American Geriatrics Society 2012 Beers Criteria Update Expert P 2012; Holt et al. 2010; Laroche et al. 2007). Table 3 shows the molecules validated by the experts through the Delphi process and considered as PIM by the literature. Of the 30 molecules present in the PDE list, 7 of them were considered as potentially inappropriate only beyond a certain daily dose; 8 could be used with caution in view of the literature and one was proposed in the form of eye drops and not for systemic use.

**Table 3:** **Drugs considered as appropriate in the elderly by the experts but inappropriate according to the literature**

ATC* classification system	Drug evaluation on the 5-point Likert scale mean and 95% confidence interval
**A. Alimentary tract and metabolism**	
Insulin, sliding scale	
Types of insulin and analogues for injection:	
Ultrafast*-*acting	1,59 [1,33; 1,85]
Fast-acting	1,60 [1,35; 1,84]
Intermediate-acting combined with fast-acting	1,62 [1,36; 1,88]
Gelified liquid paraffin	2,39 [2,01; 2,77]
Scopolamine	2,39 [2,01; 2,77]
**B. Blood and blood forming organs**	
Acetylsalicylic acid (as an antithrombotic agent)	1,41 [1,23; 1,59]
**C. Cardiovascular system**	
Amiodarone	1,81 [1,52; 2,10]
Digoxin (doses > 0,125 mg/d or [c]ser > 1.2 ng/ml)	2,10 [1,74; 2,46]
Nicardipine	2,18 [1,85; 2,52]
Sotalol	2,26 [1,99; 2,52]
Spironolactone (doses > 25 mg/d)	1,76 [1,51; 2,01]
**J. Anti-infectives for systemic use**	
Nitrofurantoin	2,45 [2,08; 2,83]
**M. Musculo-skeletal system**	
Tiemonium	2,20 [1,82; 2,57]
**N. Nervous system**	
Alprazolam (doses > 2 mg/d)	2,10 [1,81; 2,40]
Carbamazepine	2,51 [2,18; 2,84]
Citalopram	1,96 [1,71; 2,20]
Clozapine	2,38 [2,04; 2,71]
Escitalopram	1,92 [1,67; 2,17]
Loxapine	2,64 [2,31; 2,96]
Mirtazapine	2,50 [2,17; 2,82]
Olanzapine (doses > 10 mg)	2,23 [1,89; 2,56]
Oxazepam (doses > 60 mg/d)	2,04 [1,74; 2,34]
Paroxetine	1,89 [1,67; 2,11]
Piribedil	2,63 [2,28; 2,96]
Risperidone	1,98 [1,73; 2,23]
Sertraline	2,11 [1,85; 2,37]
Venlafaxine	2,27 [1,97; 2,57]
Zolpidem (doses > 5 mg/d)	2,35 [2,06; 2,65]
Zopiclone (doses > 3,5 mg/d)	2,32 [2,00; 2,63]
**S. Sensory organs**	
Indomethacin (if oral route)	2,13 [1,89; 2,37]

*ATC: Anatomic, Therapeutic and Chemical classification.

On the other hand, during the Delphi process, the experts identified 31 additional molecules as potentially inappropriate in the elderly and there has been no prior mention of these in the literature (see Table 4).

**Table 4:** **Drugs considered as inappropriate in the elderly by the experts but appropriate according to the literature**

ATC: Anatomic, Therapeutic and Chemical classification	Drug evaluation on the 5-point Likert scale Mean and 95% confidence interval			
	First round		Second round	
**A. Alimentary tract and metabolism**				
Acarbose	2,97 [2,64; 3,31]	Second round	3,27 [3,02; 3,52]	Excluded
Anethole trithione	2,88 [2,55; 3,21]	Second round	3,33 [3,07; 3,58]	Excluded
Glimepiride	2,85 [2,52; 3,17]	Second round	3,28 [3,01; 3,57]	Excluded
Magnesium aspartate	3,04 [2,69; 3,39]	Second round	3,68 [3,42; 3,94]	Excluded
Magnesium carbonate	3,02 [2,66; 3,37]	Second round	3,59 [3,31; 3,87]	Excluded
Magnesium lactate + pyridoxine	3,23 [2,90; 3,57]	Second round	3,72 [3,47; 3,97]	Excluded
Nifuroxazide	3,43 [3,12; 3,74]	Excluded		
Ranitidine	2,93 [2,60; 3,26]	Second round	3,38 [3,12; 3,64]	Excluded
Ursodeoxycholic acid	2,85 [2,48; 3,21]	Second round	3,27 [3,00; 3,54]	Excluded
**B. Blood and blood forming organs**				
Tranexamic acid	2,82 [2,47; 3,18]	Second round	3,27 [3,01; 3,53]	Excluded
**C. Cardiovascular system**				
Cibenzoline	2,97 [2,65; 3,30]	Second round	3,24 [3,02; 3,45]	Excluded
Diosmin	3,78 [3,41; 4,16]	Excluded		
Fenofibrate	3,06 [2,75; 3,38]	Second round	3,46 [3,21; 3,72]	Excluded
Trimetazidine	3,80 [3,45; 4,16]	Excluded		
**D. Dermatologicals**				
Terbinafine	2,71 [2,33; 3,08]	Second round	3,34 [3,08; 3,6]	Excluded
**G. Genito-urinary system and sex hormones**				
Neomycin + nystatin + metronidazole	3,24 [2,92; 3,56]	Second round	3,47 [3,2; 3,74]	Excluded
**H. Systemic hormonal preparations**				
Salmon calcitonin	3,13 [2,79; 3,49]	Second round	3,47 [3,2; 3,74]	Excluded
**J. Anti-infectives for systemic use**				
Telithromycin	2,92 [2,61; 3,22]	Second round	3,52 [3,28; 3,77]	Excluded
**M. Musculo-skeletal system**				
Celecoxib	3,82 [3,51; 4,14]	Excluded		
Chondroitin sulfate	3,28 [2,91; 3,65]	Second round	3,88 [3,60; 4,10]	Excluded
Dantrolene	3,23 [2,90; 3,65]	Second round	3,36 [3,11; 3,62]	Excluded
Strontium ranelate	3,35 [2,98; 3,72]	Second round	3,82 [3,55; 4,09]	Excluded
Thiocolchicoside	3,15 [2,78; 3,51]	Second round	3,38 [3,10; 3,67]	Excluded
Avocado and soybean oil	3,46 [3,08; 3,85]	Excluded		
**N. Nervous system**				
Acetaminophen + caffeine + opium	3,06 [2,74; 3,39]	Second round	3,32 [3,04; 3,59]	Excluded
Buprenorphine	3,21 [2,90; 3,52]	Second round	3,38 [3,39; 3,94]	Excluded
Pipamperone	3,48 [3,15; 3,80]			
Selegiline	3,07 [2,74; 3,39]	Second round	3,49 [3,26; 3,71]	Excluded
Tropatepine	3,32 [2,95; 3,69]	Second round	3,62 [3,34; 3,90]	Excluded
**R. Respiratory system**				
Prednisolone + naphazoline	3,44 [3,11; 3,77]	Excluded		
Theophylline	3,41 [3,06; 3,77]	Excluded		

### 3.4. Generation of the final PDE list

As mentioned above, the PDE list contains 252 drugs. A version of the list is shown in Table 5. To improve the effective use of the PDE list, valuable information for daily practices were added [the list with specific information concerning each drug is available at http://www.ars.alsace.sante.fr/Liste-preferentielle-de-medica.144691.0.html*(language: French*)]. These data were intended to guide the physician in his prescription and the nurses in drug administration. For each molecule, the existence of generic drugs was specified, all dosages were mentioned, galenic forms commonly encountered were listed (tablets, capsules, oral solutions …). For dry oral forms, the possibility of crushing the tablets or opening the capsules was described. The shelf life of oral liquid forms after first opening the container was indicated. Sugar content, sodium, potassium and ethanol were also indicated. For the 30 drugs in the PDE list considered as potentially inappropriate for the elderly by the literature, all the criteria developed in the reviews were reproduced and put forth such as arguments, clinical monitoring, therapeutic alternatives.... Warnings were indicated when the state of renal or hepatic functions needed to be taken into account for prescription.

**Table 5:** **Medication in the elderly: the preferential list of drugs obtained by consensus from a panel of 53 experts (Delphi process)**

A. Alimentary tract and metabolism	B. Blood and blood forming organs	C. Cardiovascular system	
Ascorbic acid	Acenocoumarol	Acebutolol	Nicorandil
Calcium carbonate	Acetylsalicylic acid	Amiodarone	Perindopril
Calcium carbonate + colecalciferol	Calcium heparin	Amlodipine	Perindopril + indapamide
Chlorhexidine + chlorobutanol	Clopidogrel	Atenolol	Pravastatin
Colecalciferol	Cyanocobalamin	Atorvastatin	Propranolol
Diosmectite	Enoxaparin	Benazepril + hydrochlorothiazide	Ramipril
Domperidone	Ferrous fumarate	Bisoprolol	Rosuvastatin
Ergocalciferol	Ferrous sulfate	Bumetanide	Simvastatin
Esomeprazole	Ferrous sulphate + folic acid	Candesartan	Sotalol
Gliclazide	Fluidione	Carvedilol	Spironolactone
Insulins: ultra-fast acting,	Folic acid	Celiprolol	Spironolactone + altizide
fast-acting,			
intermediate - acting intermediate-acting combined with fast-acting, long –acting			
Lactulose	Fondaparinux	Cicletanine	Valsartan
Lanzoprazole	Phytomenadione	Digoxin	Valsartan + hydrochlorothiazide
Liquid paraffin	Tinzaparin	Diltiazem	Verapamil
Macrogol	Warfarin	Enalapril	Zinc oxide + titanium dioxide + carrageenane
Macrogol in combination with potassium chloride, sodium chloride and sodium bicarbonate		Furosemide	Zinc oxide + titanium dioxide + carrageenane + lidocaine
Metformin		Glyceryl nitrate (oral)	
Omeprazole		Glyceryl nitrate (transdermal patches)	
Pancreatin		Hydrochlorothiazide	
Pantoprazole		Indapamide	
Phloroglucinol		Irbesartan	
Potassium chloride		Isosorbide dinitrate	
Psylla		Isosorbide mononitrate	
Racecadotril		Lercanidipine	
Repaglinide		Lisinopril	
**A. Alimentary tract and metabolism**		**C. Cardiovascular system**	
Scopolamine		Lisinopril + hydrochlorothiazide	
Sodium bicarbonate + alginic acid		Losartan	
Sodium bicarbonate + potassium bitartrate		Metoprolol	
Potassium gluconate		Molsidomine	
Sodium phosphate		Nebivolol	
Sorbitol		Nicardipine	
Sterculia			
**D. Dermatologicals**	**D. Dermatologicals**	**G. Genito-urinary system and sex hormones**	**H. Systemic hormonal preparations**
Aciclovir	Fusidic acid	Alfuzosin	Betamethasone
Amorolfine	Galen’s wax	Cyproterone	Carbimazole
Betamethasone	Glycerol + vaseline + liquid paraffin	Dutasteride	Glucagon
Betamethasone + salicylic acid	Hydrocortisone	Econazole	Hydrocortisone
Bifonazole	Ketoconazole	Metronidazole	Levothyroxine sodium
Calcipotriol	Povidone-iodine	Povidone-iodine	Methylprednisolone
Calcipotriol + betamethasone	Sodium hypochlorite	Promestriene	Prednisolone
Chlorhexidine + benzalkonium chloride + benzylic alcohol	Triethanolamine	Serenoa repens	Prednisone
Ciclopirox	Vaseline	Tamsulosin	
Clobetasol	Zinc oxide	Trospium	
Desonide	Zinc oxide + fish liver oil		
Econazole	Zinc oxide + glycerol + talcum powder		
**J. Anti-infectives for systemic use**	**J. Anti-infectives for systemic use**	**L. Antineoplastic and immunomodulating agents**	**M. Musculo-skeletal system**
Aciclovir	Fluconazole	Anastrozole	Alendronic acid
Amoxicillin	Fusidic acid	Bicalutamide	Alendronic acid + colecalciferol
Amoxicillin + clavulanic acid	Levofloxacin	Letrozole	Allopurinol
Amphotericin B	Metronidazole	Leuprorelin	Clodronic acid
Azithromycin	Nitrofurantoin	Tamoxifen	Colchicine + tiemonium + opium
**J. Anti-infectives for systemic use**	**J. Anti-infectives for systemic use**		**M. Musculo-skeletal system**
Cefixime	Norfloxacin		Diclofenac (topic use)
Cefpodoxime	Ofloxacin		Diclofenac (oral use)
Ceftriaxone	Oseltamivir		Ibuprofen
Cefuroxim	Pristinamycin		Risedronic acid
Ciprofloxacin	Roxithromycin		
Clarithromycin	Spiramycin		
Cloxacillin Doxycycline	Sulfamethoxazole + trimethoprim		
	Valaciclovir		
**N. Nervous system**	**N. Nervous system**	**R. Respiratory system**	**S. Sensory organs**
Acetaminophen	Mirtazapine	Acetylcysteine	Acetazolamide
Alprazolam	Nefopam	Beclometasone	Artificial tears
Carbamazepine	Olanzapine	Budesonide	Carbomers
Citalopram	Oxazepam	Carbocisteine	Dexamethasone + oxytetracycline
Clozapine	Paroxetine	Desloratadine	Indometacin
Codeine + acetaminophen	Piribedil	Fenoterol + ipratropium	Latanoprost
Donepezil	Pramipexole	Formoterol	Ofloxacin
Entacapone	Pregabalin	Helicidine	Pilocarpine
Escitalopram	Risperidone	Ipratropium bromide	Retinol
Fentanyl	Rivastigmine	Levocetirizine	Rifamycin
Gabapentin	Ropinirole	Loratadine	Timolol
Galantamine	Sertraline	Montelukast	Xylene
Hydrochloride morphine	Fast –acting Morphine sulfate	Salbutamol	
Lamotrigine	Long-actingMorphine sulfate	Salmeterol + fluticasone	
Levodopa + decarboxylase inhibitor	Tiapride	Terbutaline	
Levodopa + decarboxylase inhibitor + COMT inhibitor	Tramadol + acetaminophen	Tiotropium bromide	
Lidocaine	Valproic acid	Tixocortol	
Lidocaine + prilocaine	Valpromide		
Lithium	Venlafaxine		
Loxapine	Zolpidem		
Memantine	Zopiclone		
Mianserin			

The detailed list is available at http://www.ars.alsace.sante.fr/Liste-preferentielle-de-medica.144691.0.html *(language: French*); presentation of drugs according to ATC: Anatomic, Therapeutic and Chemical classification.

## 4. Discussion

We have proposed a preferential list of drugs adapted to the elderly in nursing homes (PDE list) that includes molecules and their presentation forms having a utility, a favorable balance risk/benefit or a well-established use in this particular population. This list is based upon methods that included : i) creating a preliminary list from drug formularies used in daily practices in nursing homes in Alsace, ii) soliciting experts through a Delphi process, iii) identifying molecules considered in literature as PIM. Thus, the PDE list is the result of multifaceted interventions to achieve optimal results for prescribing drugs for elderly people.

The field for optimization of drug prescriptions in the elderly has received great attention in the last few years. A range of strategies has been implemented to precisely define inappropriate practices in older people (Topinkova et al. 2012). For example, efforts have been made in the USA, in Germany and in France to identify PIMs among drugs that are available in each of these countries (The American Geriatrics Society 2012 Beers Criteria Update Expert P 2012; Holt et al. 2010; Laroche et al. 2007) in order to be used with caution. The best known screening tool to reduce inappropriate prescribing is the Beers list which has been recently updated by the American geriatrics society (The American Geriatrics Society 2012 Beers Criteria Update Expert P 2012). These lists can also be called “negative lists”. By contrast, the issue of appropriate medication *per se* is poorly documented. The use of lists containing drugs with a benefit-to-risk ratio acceptable in the elderly is rarely proposed as an approach that can be used to ensure the appropriateness of prescribing, and as far as we know, our PDE list constitutes the first data ever published in France.

The methodology used for building the PDE list began with an inventory of the available drug formularies used in nursing homes in Alsace. Eleven drug formularies were analyzed, 591 drugs were counted. The comparison of the content of these formularies and the data obtained from Alsatian general health insurance concerning reimbursed drugs for the elderly authorized us to state that these 591 drugs were representative of those used in the whole region.

For practical reasons, all the molecules were not reviewed by experts. If the drug was present in 20% of the analyzed formularies- 20% thus being set as the arbitrary cut-off percentage- it was then included to be part of the preliminary list. The main argument for the determination of the deciding percentage was based on the idea that a molecule present in only one or two formularies responded to needs associated with specific care management.

The Delphi method is a consensus technique used and validated in various health domains such as various clinical practices (Jones & Hunter 1995). This approach allowed us to submit the preliminary list to a panel of experts. The subjectivity of the assessment by a consensus of experts is obvious, but can be overcome by requesting a large number of experts. In our case, 48 experts participated in the first round of questioning, and 53 completed the second round. Moreover, the PDE list combined this data with the opinion of practitioners commonly involved in the management of drugs given to elderly patients. These experts represented different specialties and were from different parts of France and Europe, in order to give a large overview of the practices. Finally, experts were consulted separately, hence they were not able to discuss together but were capable of adding commentaries in order to communicate their ideas between both rounds. This approach allowed experts to express their opinions independently and confidentially without any peer-pressure or conflicts of judgement that may occur during a face-to-face meeting. Therefore, we are confident that drugs listed here, are definitely regarded as useful in daily practices by a large and diverse group of specialists.

In our study, 30 drugs from the PDE list were considered as potentially inappropriate in view of the published studies (The American Geriatrics Society 2012 Beers Criteria Update Expert P 2012; Holt et al. 2010; Laroche et al. 2007). We however decided to keep these drugs in the PDE list and decided to clearly identify them as potentially inappropriate as a reminder for closer monitoring of these 30 drugs. On the other hand, experts in our study excluded 31 molecules which are not cited in the literature as inappropriate. Some of them have restricted indications or seem to be unnecessary in nursing homes: tranexamic acid, calcitonin, dantrolene, ursodeoxycholic acid, cibenzoline are examples. Others present a lack of interest: avocado and soybean oil, chondroitin sulfate, magnesium. Finally, some molecules present an unfavorable benefit-to-risk ratio: terbinafine, theophylline, ranitidine, strontium ranelate and acarbose. The differences between the published PIM lists and our results could reflect the use of different methodological approaches, the subjectivity of assessments obtained by consensus among experts, the pharmaceutical supply available in different countries. It should also be noted that significant differences exist between the PIM lists published so far (The American Geriatrics Society 2012 Beers Criteria Update Expert P 2012; Holt et al. 2010; Laroche et al. 2007).

Concerning the administration of drugs in the elderly, as mentioned by Caussin et al. (Caussin et al. 2012), there is an enormous potential for improvement in drugs safety and effectiveness. In geriatrics, it is frequent to crush pills or simply open capsules so that patients presenting problems swallowing and/or behavior issues may take the medicines more easily. In the study mentioned above, 42% of crushed drugs had a galenic presentation which did not allow crushing. These practices, marked by frequent errors, may significantly alter the effectiveness of drugs, their pharmacokinetics and even could lead to toxic effects for both patients and caregivers. All classes of drugs are concerned. To avoid these potential iatrogenic and professional risks, the PDE list indicates clearly which drugs should not be crushed and, in those cases, suggests alternative measures.

Finally, the PDE list constitutes a general guide for the optimization of both prescription and administration of drugs in nursing homes and this could help reduce misuses and poly-medication, both of which are constant preoccupations to avoid ADRs.

Nevertheless, this PDE list has some limitations. First of all, it is important to concede that the PDE list was built from Alsatian data. However, physicians from other French regions should not encounter any major problems adopting this tool in their nursing homes. Solicited experts belong to national professionals and scientific societies, some of them coming from different other regions of France. This guaranteed that local prescribing practices did not overly influence the development of the list. On the other hand, the PDE list has limited applicability for international use. Country-specific prescribing trends, disease epidemiology, differences in drug availability must not be ignored.

Secondly, the PDE list is focused on medication in nursing homes. The extension of the study to elderly in ambulatory care could be viewed as one of the next important steps in the updating of the PDE list.

Thirdly, this PDE list should not be used without adequate clinical expertise. For a given patient, a benefit-to-risk ratio for each drug has to be assessed considering clinical conditions, comorbidities, functional status, other drugs taken and prognosis. Further, the issue of prescribing appropriateness, in its broader sense, must encompass steps in favor of non-drug approaches (beneficial drug omission) and include also patients’ preferences to achieve optimal results (Spinewine et al. 2007).

Fourthly, the PDE list could be controversial because of the limitations imposed by this list to the prescribing physician. It can be argued that the PDE list is a starting tool. Specific adaptations conducted within each nursing home in collaboration with physicians, pharmacist, nurses, and possibly administrative directors, are a suitable response to allow the intelligent and judicious adoption of the PDE list.

## 5. Conclusion

Establishing a list of drugs to be used preferentially in nursing homes is written in the French Code of Public Health (articles L. 313–12 and L. 5126-6-1). It has to be developed in each nursing home in a multidisciplinary context by including coordinating physician, pharmacist and general practitioners. As we can see in this article only few Alsatian nursing homes have developed their own formularies.

The PDE list constitutes a unique and starting guideline and can by no means be enforced by law but it can be used mostly to harmonize practices in nursing homes and to help physicians and nurses to achieve best possible care management. We expect that the PDE list will have to be regularly reviewed to be fully useful to health professionals. A further challenge to facilitate the adoption of the PDE list will be to demonstrate that implementation of this tool, adjusted to the needs of nursing homes, will result in objective and quantifiable improvements in the management of older people. We are currently completing a randomized controlled study among 10 nursing homes in Alsace to assess these issues in terms of clinical and economic outcomes.
